# Nucleus accumbens core lesions retard instrumental learning and performance with delayed reinforcement in the rat

**DOI:** 10.1186/1471-2202-6-9

**Published:** 2005-02-03

**Authors:** Rudolf N Cardinal, Timothy HC Cheung

**Affiliations:** 1 Department of Experimental Psychology, University of Cambridge, Downing Street, Cambridge CB2 3EB, UK; 2 Psychopharmacology Section, Division of Psychiatry, B Floor, Medical School, Queen's Medical Centre, Nottingham NG7 2UH, UK

## Abstract

**Background:**

Delays between actions and their outcomes severely hinder reinforcement learning systems, but little is known of the neural mechanism by which animals overcome this problem and bridge such delays. The nucleus accumbens core (AcbC), part of the ventral striatum, is required for normal preference for a large, delayed reward over a small, immediate reward (self-controlled choice) in rats, but the reason for this is unclear. We investigated the role of the AcbC in learning a free-operant instrumental response using delayed reinforcement, performance of a previously-learned response for delayed reinforcement, and assessment of the relative magnitudes of two different rewards.

**Results:**

Groups of rats with excitotoxic or sham lesions of the AcbC acquired an instrumental response with different delays (0, 10, or 20 s) between the lever-press response and reinforcer delivery. A second (inactive) lever was also present, but responding on it was never reinforced. As expected, the delays retarded learning in normal rats. AcbC lesions did not hinder learning in the absence of delays, but AcbC-lesioned rats were impaired in learning when there was a delay, relative to sham-operated controls. All groups eventually acquired the response and discriminated the active lever from the inactive lever to some degree. Rats were subsequently trained to discriminate reinforcers of different magnitudes. AcbC-lesioned rats were more sensitive to differences in reinforcer magnitude than sham-operated controls, suggesting that the deficit in self-controlled choice previously observed in such rats was a consequence of reduced preference for delayed rewards relative to immediate rewards, not of reduced preference for large rewards relative to small rewards. AcbC lesions also impaired the performance of a previously-learned instrumental response in a delay-dependent fashion.

**Conclusions:**

These results demonstrate that the AcbC contributes to instrumental learning and performance by bridging delays between subjects' actions and the ensuing outcomes that reinforce behaviour.

## Background

Animals learn to control their environment through instrumental (operant) conditioning. When an animal acts to obtain reward or reinforcement, there is often a delay between its action and the outcome; thus, animals must learn instrumental action-outcome contingencies using delayed reinforcement. Although such delays impair learning, animals can nevertheless bridge substantial delays to acquire instrumental responses [1]. Little is known of the neural basis of this process. However, abnormalities in learning from delayed reinforcement may be of considerable clinical significance [2]. Impulsivity is part of the syndrome of many psychiatric disorders, including mania, drug addiction, antisocial personality disorder, and attention-deficit/hyperactivity disorder [3]. Impulsive choice, one aspect of impulsivity [4], is exemplified by the tendency to choose small rewards that are available immediately instead of larger rewards that are only available after a delay [5,6], and may reflect dysfunction of reinforcement learning systems mediating the effects of delayed rewards [5,7].

The nucleus accumbens (Acb) responds to anticipated rewards in humans, other primates, and rats [8-15], and is innervated by dopamine (DA) neurons that respond to errors in reward prediction in a manner appropriate for a teaching signal [16-19]. The Acb may therefore represent a reinforcement learning system specialized for learning with delayed reinforcement [20,21]. If this is the case, then damage to the Acb should not interfere with reinforcement learning in all circumstances, but should produce selective impairments in learning when reinforcement is delayed. This prediction has not previously been tested. However, lesions of the AcbC cause rats to prefer small immediate rewards (a single food pellet delivered immediately) to large delayed rewards (four pellets delivered after a delay); that is, AcbC-lesioned rats exhibit impulsive choice [22,23]. The reason for this is not clear. It might be that AcbC-lesioned rats exhibit steeper temporal discounting, such that the subjective utility (value) of future rewards declines more rapidly than normal as the reward is progressively delayed [24,25]. It might also be that AcbC-lesioned rats are less good at representing the contingency between actions and their outcomes when the outcomes are delayed, so that they choose impulsively because they are less certain or less aware that their choosing the delayed reward does in fact lead to that reward being delivered [24,25]. Both explanations would reflect a problem in dealing with delayed reinforcement in AcbC-lesioned rats. However, there might be a simpler explanation for the impulsive choice exhibited by AcbC-lesioned rats: they might perceive the size (magnitude) of rewards differently. For example, if they do not perceive the delayed reward to be as large, relative to the immediate reward, as normal rats did, then they might choose impulsively despite processing the delays to reward normally, simply because the delayed reinforcer is not subjectively large enough to compensate for the normal effects of the delay [24-26].

**Figure 1:**
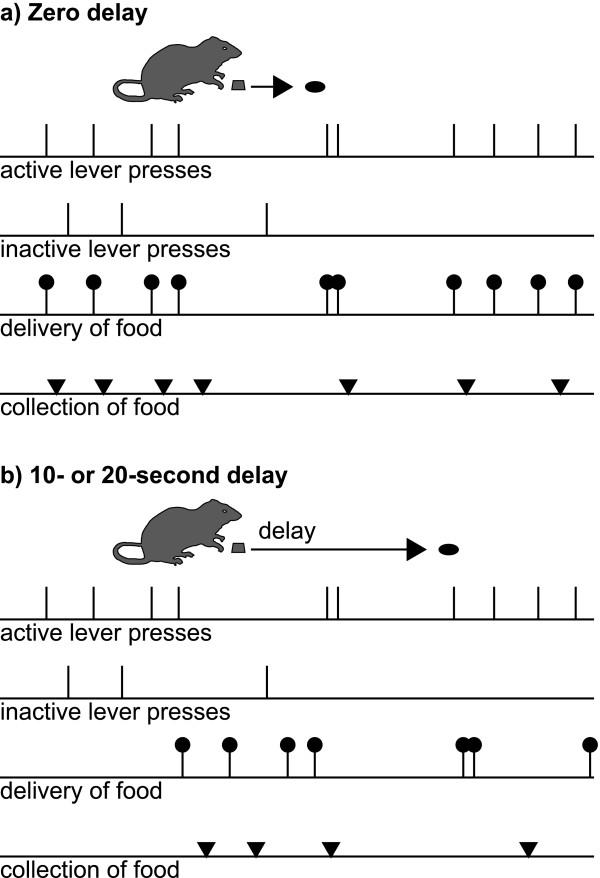
**Task schematic: free-operant instrumental responding on a fixed-ratio-1 (FR-1) schedule with delayed reinforcement **Subjects are offered two levers; one (the active lever) delivers a single food pellet for every press (an FR-1 schedule) and the other (the inactive lever) has no programmed consequence. Food can either be delivered immediately **(a) **or after a delay **(b) **following responses on the active lever. The levers remain available throughout the session (hence, free-operant responding: animals are free to perform the operant at any time). Events of interest are lever presses, delivery of food pellets, and collection of food by the rat (when it pokes its nose into the food alcove following food delivery). To obtain food, the hungry rat must discriminate the active from the inactive lever, which is more difficult when the outcome is delayed. In these examples, the rat's response patterns (active and inactive lever presses, and collection of food) are fictional, while food delivery is contingent upon active lever pressing.

To investigate whether the AcbC is a reinforcement learning system specialized for delayed reinforcement, we first determined the ability of AcbC-lesioned rats to detect instrumental contingencies across a delay. The ability of AcbC-lesioned rats to acquire instrumental responding with delayed reinforcement was compared to that of sham-operated controls; each subject was allowed to respond freely on two levers, one of which produced reinforcement after a delay of 0, 10, or 20 s (Figure 1). We report that AcbC lesions only retarded instrumental learning when reinforcement was delayed, demonstrating a role for the AcbC in bridging action-outcome delays during learning. Subsequently, to establish whether AcbC-lesioned rats perceive reward magnitude abnormally, we assessed these subjects' sensitivity to reinforcer magnitude by measuring their relative preference for two different reinforcers using concurrent interval schedules of reinforcement. We report that reinforcer magnitude discrimination in AcbC-lesioned rats in this task was at least as good as in sham-operated controls, consistent with previous evidence of reinforcer magnitude discrimination following lesions of the whole Acb e.g. [27,28]. Together, these results suggest that the impulsive choice seen in AcbC-lesioned rats [22] is due to a problem in processing delayed reward, not in processing the magnitudes of the reward alternatives. Finally, to establish whether the AcbC is required for the performance of an instrumental response for delayed reinforcement, as well as for the learning of such a response, we trained naïve rats to respond for delayed reinforcement (Figure 1) before destroying the AcbC. We report that such lesions also impaired performance of a previously-learned instrumental response only when reinforcement was delayed, indicating that the AcbC makes an enduring contribution to bridging delays between subjects' actions and the ensuing outcomes.

## Results

In Experiment 1, rats received excitotoxic lesions of the AcbC or sham lesions, and were then tested on an instrumental free-operant acquisition task with delayed reinforcement (Experiment 1A; see Methods) and subsequently a reinforcer magnitude discrimination task (Experiment 1B). In Experiment 2, naïve rats were trained on the free-operant task for delayed reinforcement; AcbC lesions were then made and the rats were retested.

### Histology

In Experiment 1, there were two postoperative deaths. Histological analysis revealed that the lesions were incomplete or encroached significantly on neighbouring structures in four subjects. These subjects were excluded; final group numbers were therefore 8 (sham, 0 s delay), 6 (AcbC, 0 s delay), 8 (sham, 10 s delay), 7 (AcbC, 10 s delay), 8 (sham, 20 s delay), and 7 (AcbC, 20 s delay). In Experiment 2, one rat spontaneously fell ill with a colonic volvulus during preoperative training and was killed, and there were three postoperative deaths. Lesions were incomplete or too extensive in seven subjects; final group numbers were therefore 7 (sham, 0 s delay), 5 (AcbC, 0 s delay), 8 (sham, 10 s delay), 4 (AcbC, 10 s delay), 8 (sham, 20 s delay), and 5 (AcbC, 20 s delay).

**Figure 2:**
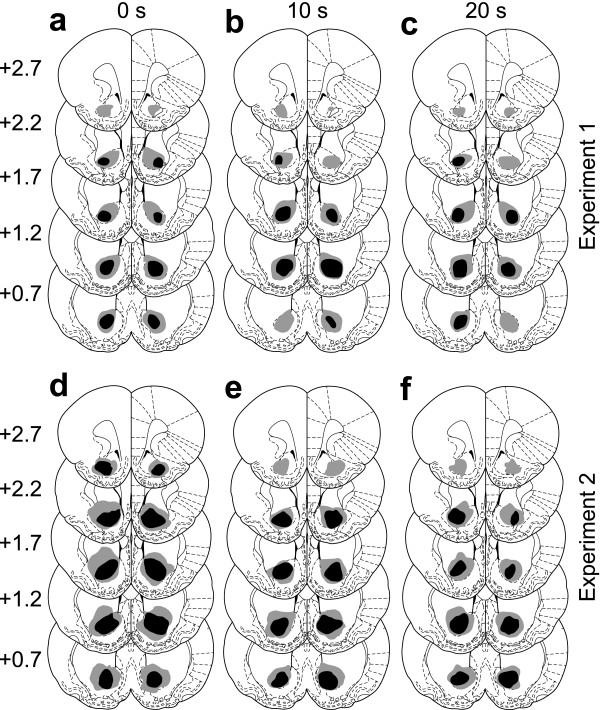
**Schematic of lesions of the AcbC **Black shading indicates the extent of neuronal loss common to all subjects; grey indicates the area lesioned in at least one subject. Coronal sections are (from top to bottom) +2.7, +2.2, +1.7, +1.2, and +0.7 mm relative to bregma. Diagrams are modified from reference [83]. Panels **a-c **correspond to Experiment 1, in which lesions were made before training; panels **d-f **correspond to Experiment 2, in which lesions were made after initial training. Panels **a & d **show groups trained with no delays; panels **b & e **show groups trained with 10 s delays; panels **c & f **show groups trained with 20 s delays.

**Figure 3:**
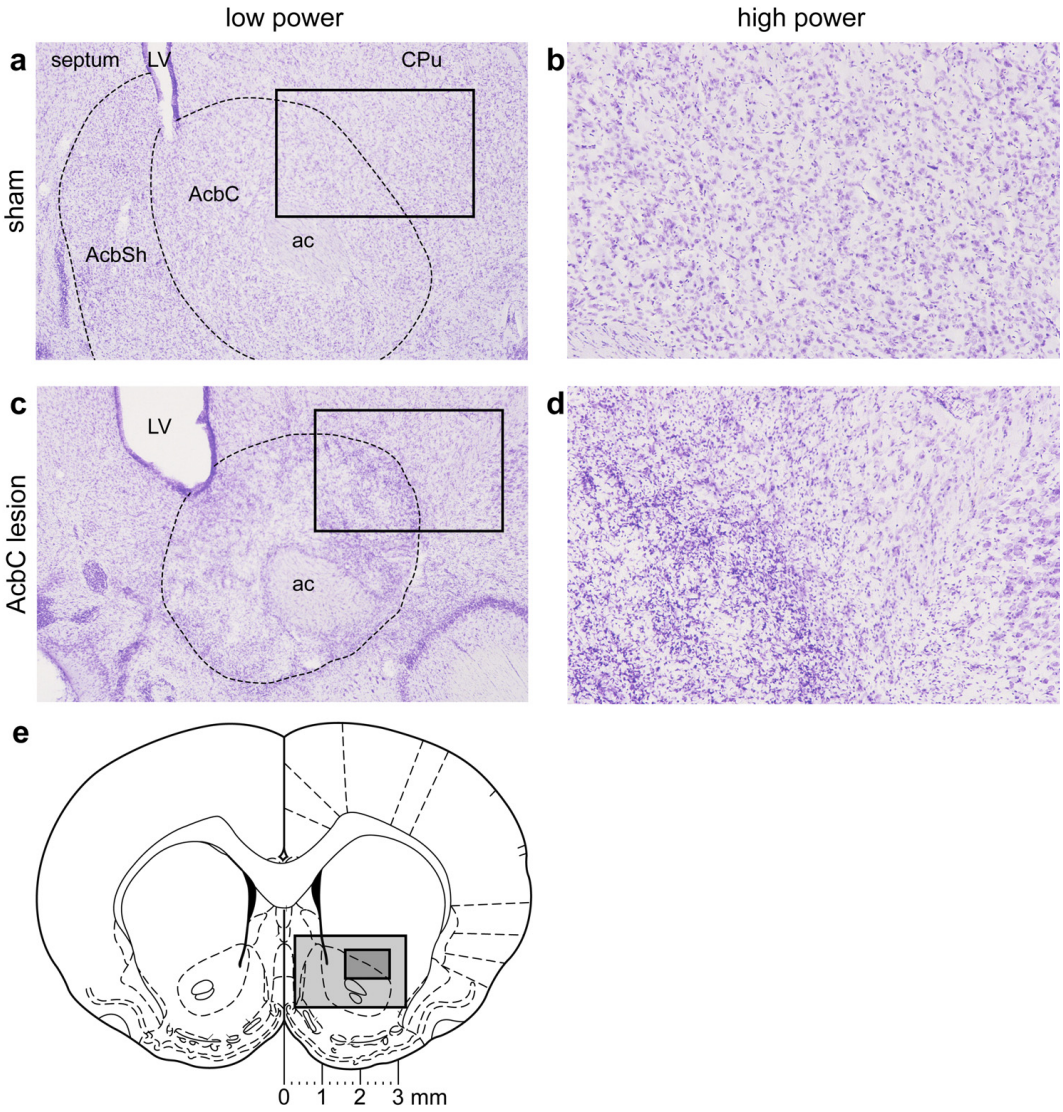
**Photomicrographs of lesions of the AcbC **Lesions of the AcbC: photomicrographs of sections ~1.2 mm anterior to bregma, stained with cresyl violet. **(a) **Sham-operated rat, low-magnification view, right hemisphere (medial to the left). LV, lateral ventricle; CPu, caudate/putamen; AcbSh, nucleus accumbens shell; AcbC, nucleus accumbens core; ac, anterior commissure. The box marks the area magnified in (b). **(b) **Sham-operated rat, high-magnification view. Cresyl violet is basic and stains for Nissl substance, primarily nucleic acids (DNA and RNA); it therefore stains cytoplasmic rough endoplasmic reticulum, nuclei, and nucleoli. Individual neuronal nuclei are visible (circles ~10 μm in diameter). **(c) **AcbC-lesioned rat, low-magnification view. Dotted lines show the approximate extent of the lesion. There is some tissue collapse within the lesion and the lateral ventricle is slightly expanded. The box marks the area magnified in (d). **(d) **AcbC-lesioned rat, high-magnification view. In the region of the lesion, neurons have been replaced by smaller, densely-staining cells, indicating gliosis. **(e) **Coronal diagram of the rat brain at the same anteroposterior level [83], with scale. The light grey box indicates approximately the region shown in (a) and (c); the dark grey box indicates approximately the region shown in (b) and (e).

Lesions of the AcbC encompassed most of the core subregion; neuronal loss and associated gliosis extended in an anteroposterior direction from approximately 2.7 mm to 0.5 mm anterior to bregma, and did not extend ventrally or caudally into the ventral pallidum or olfactory tubercle. Damage to the ventromedial caudate-putamen was occasionally seen; damage to AcbSh was restricted to the lateral edge of the dorsal shell. Schematics of the lesions are shown in Figure 2. Photomicrographs of one lesion are shown in Figure 3, and are similar to lesions with identical parameters that have been presented before [29,30].

### Acquisition of instrumental responding (Experiment 1A)

**Figure 4:**
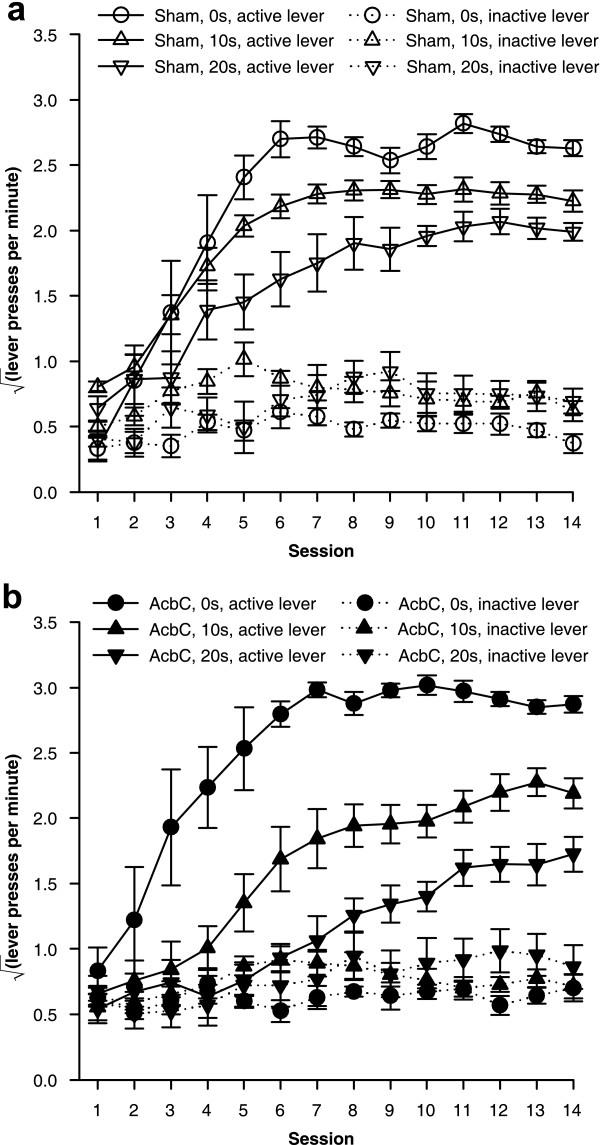
**Effects of delays to reinforcement on acquisition of free-operant responding under an FR-1 schedule **Data plotted to show the effects of delays. All groups discriminated between the active and the inactive lever, and delays retarded acquisition of the active lever response in both groups. **(a) **Responding of sham-operated control rats, under all three response-reinforcer delay conditions. **(b) **Responding of AcbC-lesioned rats under all delay conditions. The next figure replots these data to show the effect of the lesion more clearly.

**Figure 5:**
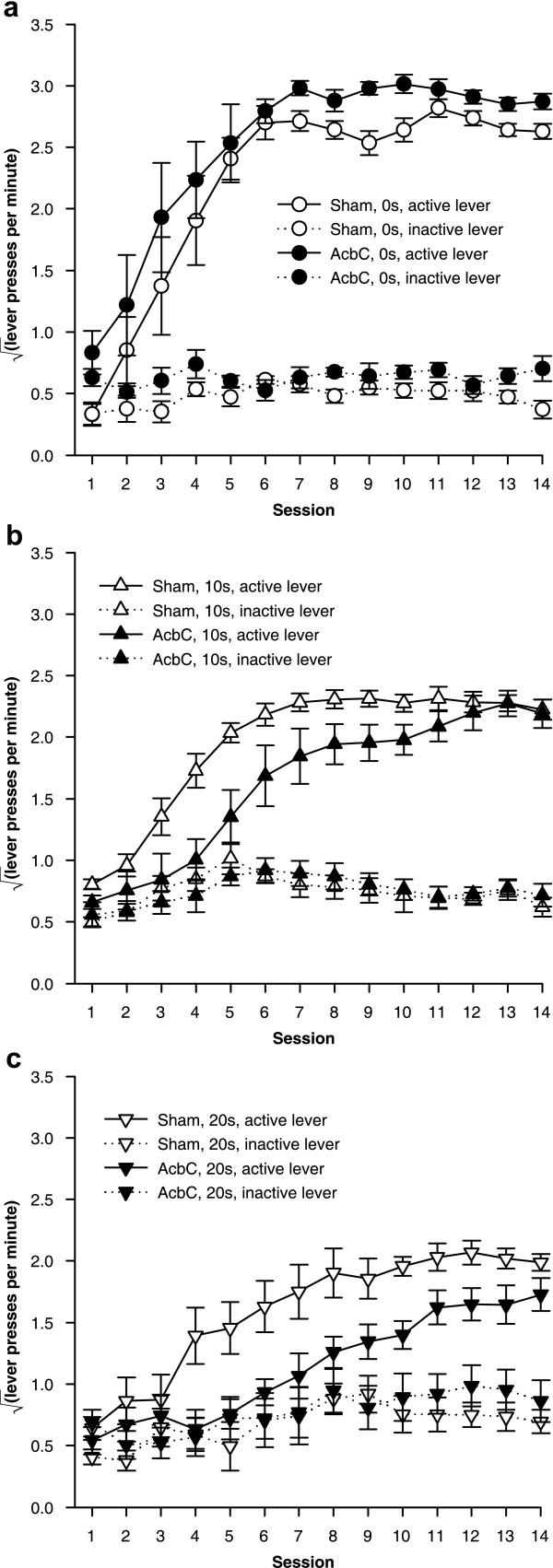
**Effect of AcbC lesions on acquisition of free-operant responding with delayed reinforcement **Data plotted to show the effects of AcbC lesions (same data as in the previous figure). There was a delay-dependent impairment in AcbC-lesioned rats, who learned less well than shams only when reinforcement was delayed. **(a) **With a delay of 0 s, AcbC-lesioned rats learned just as well as shams; in fact, they responded more on the active lever than shams did. **(b) **With a 10 s delay, AcbC-lesioned rats were impaired at learning compared to shams. **(c) **With a 20 s delay, the impairment in AcbC-lesioned rats was larger still.

The imposition of response-reinforcer delays retarded the acquisition of free-operant lever pressing, in sham-operated rats and in AcbC-lesioned rats (Figure 4). AcbC-lesioned rats responded slightly more than shams on both the active and inactive levers in the absence of response-reinforcers delays, but when such delays were present, AcbC lesions retarded acquisition relative to sham-operated controls (Figure 5).

An overall ANOVA using the model lesion_2 _× delay_3 _× (session_14 _× lever_2 _× S) revealed multiple significant interactions, including lever × delay × lesion (*F*_2,38 _= 5.17, *p *= .01) and session × lever × delay (*F*_6.0,229.1 _= 5.47,  = .464, *p *< .001), justifying sub-analysis. All six groups learned to respond more on the active lever than the inactive lever (*p *≤ .002, main effect of lever or session × lever interaction for each group alone).

For sham-operated rats, delays reduced the rate of acquisition of the active lever response and reduced the asymptotic level of responding attained (Figure 4a; delay: *F*_2,21 _= 11.7, *p *< .001;  = .276, *p *< .001; session × delay: *F*_7.2,75.3 _= 2.46,  = .276, *p *= .024). The presence of a delay also increased responding on the inactive lever slightly (delay: *F*_2,21 _= 4.06, *p *= .032), though not systematically (the 10 s group differed from the 0 s group, *p *= .036, but no other groups differed, *p *≥ .153).

There was a further, delay-dependent impairment in AcbC-lesioned rats, who responded more than shams at 0 s delay but substantially less than shams at 10 s and 20 s delay. As in the case of sham-operated controls, delays reduced the rate of acquisition and the maximum level of responding attained in AcbC-lesioned rats (Figure 4b; delay: *F*_2,17 _= 54.6, *p *< .001; delay × session: *F*_6.9,58.7 _= 2.64,  = .266, *p *= .02). Responding on the inactive lever was not significantly affected by the delays (maximum *F*_15.8,134.2 _= 1.65,  = .607, *p *= .066). At 0 s delay, AcbC-lesioned subjects responded more than shams on the active lever (Figure 5a; lesion: *F*_1,12 _= 5.30, *p *= .04) and the inactive lever (lesion: *F*_1,12 _= 9.12, *p *= .011). However, at 10 s delay, AcbC-lesioned rats responded significantly less than shams on the active lever (Figure 5b; lesion: *F*_1,13 _= 9.04, *p *= .01); there was no difference in responding on the inactive lever (*F *< 1, NS). At 20 s delay, again, AcbC-lesioned rats responded significantly less than shams on the active lever (Figure 5c; lesion: *F*_1,13 _= 9.87, *p *= .008) and there was no difference in responding on the inactive lever (*F *< 1, NS).

### Experienced response-delivery and response-collection delays (Experiment 1A)

For every reinforcer delivered, the active lever response most closely preceding it in time was identified, and the time between that response and delivery of the reinforcer (the 'response-delivery delay') was calculated. This time can therefore be equal to or less than the programmed delay, and is only relevant for subjects experiencing non-zero programmed response-reinforcer delays. The response-to-reinforcer-collection ('response-collection') delays were also calculated: for every reinforcer delivered, the response most closely preceding it and the nosepoke most closely following it were identified, and the time between these two events calculated. This time can be shorter or longer than the programmed delay, and is relevant for all subjects.

**Figure 6:**
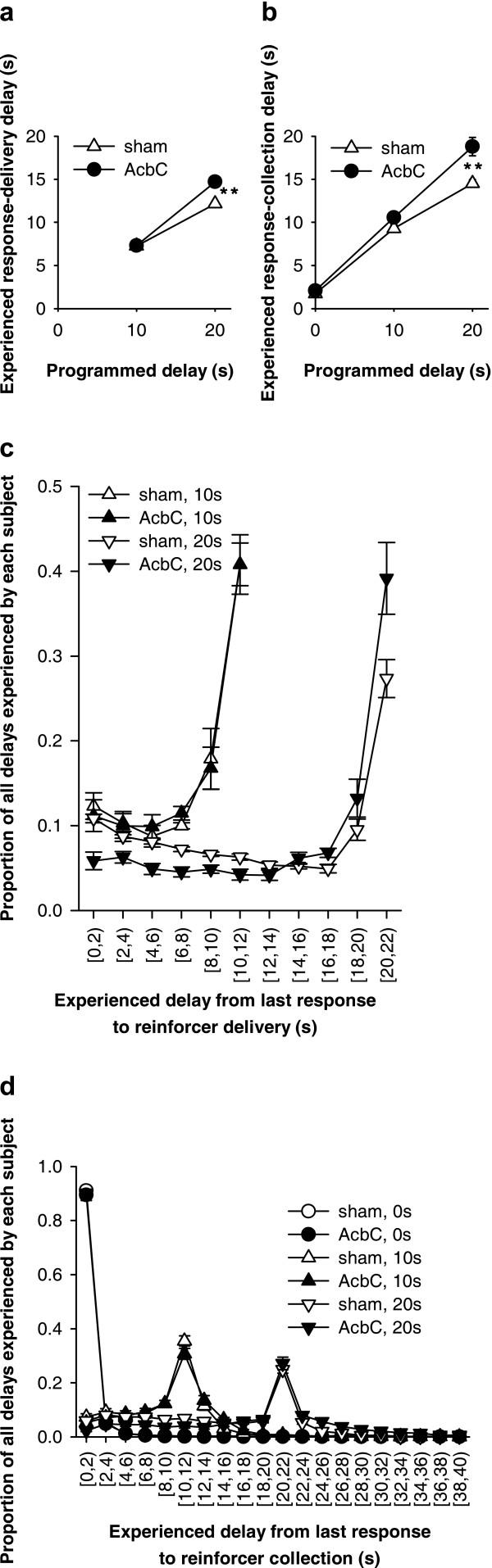
**Programmed and experienced delays to reinforcement **AcbC-lesioned rats experienced slightly longer response-delivery delays (the delay between the most recent active lever press and pellet delivery) than shams in the 20 s condition, and slightly longer response-collection delays (the delay between the most recent active lever press and pellet collection) in the 10 s and 20 s conditions. **(a) **Mean experienced response-delivery delays (one value calculated per subject). When the programmed delay was 0 s, reinforcers were delivered immediately so no data are shown. There was a lesion × programmed delay interaction (*F*_1,26 _= 12.0, *p *= .002): when the programmed delay was 10 s, the experienced delays did not differ between groups (*F *< 1, NS), but when the programmed delay was 20 s, AcbC-lesioned rats experienced longer response-delivery delays (one-way ANOVA, *F*_1,13 _= 19.0, ** *p *= .001). **(b) **Mean experienced response-collection delays (one value calculated per subject). There was a lesion × programmed delay interaction (*F*_2,38 _= 7.14, *p *= .002): AcbC-lesioned rats did not experience significantly different delays when the programmed delay was 0 s (*F *< 1, NS) or 10 s (*F*_1,13 _= 4.52, *p *= .053), but experienced significantly longer response-collection delays when the programmed delay was 20 s (*F*_1,13 _= 15.4, ** *p *= .002). **(c) **Distribution of experienced response-delivery delays. All experienced delays for a given subject were aggregated across all sessions, and the proportion falling into different 2 s ranges were calculated to give one value per range per subject; the graphs show means ± SEMs of these values. The interval notation '[*a*, *b*)' indicates that a given delay *x *falls in the range *a *≤ *x *<*b*. There were no differences in the distribution of delays experienced by AcbC-lesioned and sham rats in the 10 s condition (lesion and lesion × range, *F*s < 1, NS), but in the 20 s condition AcbC-lesioned rats experienced slightly fewer short delays and slightly more long delays (lesion × range, *F*_2.1,27.7 _= 6.60,  = .213, *p *= .004). **(d) **Distribution of experienced response-collection delays, displayed in the same manner as (c). There were no differences in the distribution of delays experienced by AcbC-lesioned and sham rats in the 0 s condition (lesion and lesion × range, *F*s < 1, NS). In the 10 s condition, AcbC-lesioned rats experienced a slightly higher proportion of long response-collection delays and a slightly lower proportion of short response-collection delays (lesion, *F*_1,13 _= 6.36, *p *= .036, though the lesion × range interaction was not significant, *F*_2.6,34.3 _= 1.74,  = .139, *p *= .181). Similarly, in the 20 s condition, AcbC-lesioned rats experienced a slightly higher proportion of long response-collection delays and a slightly lower proportion of short response-collection delays than shams (lesion × range, *F*_4.2,54.8 _= 6.65,  = .222, *p *< .001).

AcbC-lesioned rats experienced the same response-delivery delays as shams when the programmed delay was 10 s, but experienced longer response-delivery delays when the programmed delay was 20 s (Figure 6a). Similarly, AcbC-lesioned rats experienced the same response-collection delays as shams when the programmed delay was 0 s, slightly but not significantly longer response-collection delays when the programmed delay was 10 s, and significantly longer response-collection delays when the programmed delay was 20 s (Figure 6b). These differences in the *mean *delay experienced by each rat were reflected in differences in the *distribution *of response-delivery and response-collection delays when the programmed delay was non-zero (Figure 6c,d). Since AcbC-lesioned rats experienced slightly longer delays than sham-operated rats, it was necessary to take this into account when establishing the effect of delays on learning, as follows.

### Effect of delays on learning (Experiment 1A)

**Figure 7:**
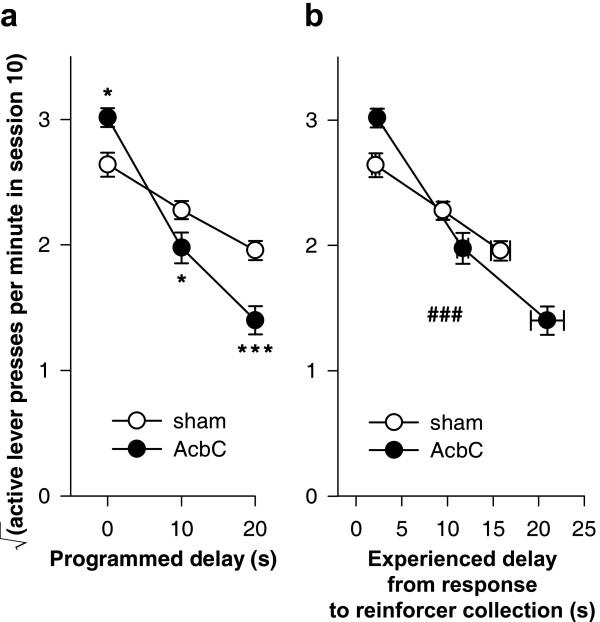
**Learning as a function of programmed and experienced delays to reinforcement **The imposition of response-reinforcer delays systematically retarded the acquisition of free-operant instrumental responding, and this relationship was altered in AcbC-lesioned rats, even allowing for differences in experienced response-collection delays. **(a) **The rate of responding on the active lever in session 10 is plotted against the programmed response-reinforcer delay. AcbC-lesioned rats responded more than shams at zero delay (* *p *= .013), but less than shams at 10 s (* *p *= .049) and 20 s delay (*** *p *= .001). **(b) **Responding on the active lever in session 10 plotted against the experienced response-to-reinforcer collection delays for sessions 1–10 (vertical error bars: SEM of the square-root-transformed number of responses in session 10; horizontal error bars: SEM of the experienced response-collection delay, calculated up to and including that session). The gradients of the two lines differed significantly (### *p *= .001; see text), indicating that the relationship between experienced delays and responding was altered in AcbC-lesioned rats.

There was a systematic relationship between the acquisition rate and the programmed delay of reinforcement, and this was altered in AcbC-lesioned rats. Figure 7a replots the rates of responding on the active lever on session 10 of acquisition [1]. Despite the comparatively low power of such an analysis, lever-pressing was analysed for this session only using the model lesion_2 _× delay_3_. This revealed a significant lesion × delay interaction (*F*_2,38 _= 12.6, *p *< .001), which was analysed further. Increasing delays significantly reduced the rate of responding in this session for shams (*F*_2,21 _= 17.3, *p *< .001) and AcbC-lesioned rats (*F*_2,17 _= 54.4, *p *< .001). AcbC-lesioned rats responded more than shams at zero delay (*F*_1,12 _= 8.52, *p *= .013) but less than shams at 10 s delay (*F*_1,13 _= 4.71, *p *= .049) and at 20 s delay (*F*_1,13 _= 17.3, *p *= .001).

Since the AcbC group experienced slightly longer response-delivery and response-collection delays than shams when the programmed delay was non-zero (Figure 6), it was important to establish whether this effect alone was responsible for the retardation of learning, or whether delays retarded learning in AcbC-lesioned rats over and above any effect to increase the experienced delay. The mean experienced response-collection delay was calculated for each subject, up to and including session 10. The square-root-transformed number of responses on the active lever in session 10 was then analysed using a general linear model of the form lesion_2 _× experienced delay_cov_. Unlike a standard analysis of covariance, the factor × covariate interaction term was included in the model. This confirmed that the lesion retarded the acquisition of responding in AcbC-lesioned rats, compared to controls, in a delay-dependent manner, over and above the differences in experienced delay (Figure 7b; lesion × experienced delay: *F*_1,40 _= 12.4, *p *= .001).

### Experienced delays and learning on the inactive lever (Experiment 1A)

No such delay-dependent effects were observed for the inactive lever. Experienced inactive-response-delivery delays (calculated across all sessions in the same manner as for the active lever) were much longer and more variable than corresponding delays for the active lever, because subjects responded on the inactive lever so little. Means ± SEMs were 250 ± 19 s (sham, 0 s), 214 ± 29 s (AcbC, 0 s), 167 ± 23 s (sham, 10 s), 176 ± 33 s (AcbC, 10 s), 229 ± 65 s (sham, 20 s), and 131 ± 37 s (AcbC, 20 s). ANOVA of these data revealed no effects of lesion or programmed delay and no interaction (maximum *F*_1,38 _= 1.69, NS). Experienced inactive-response-collection delays were 252 ± 19 s (sham, 0 s), 217 ± 29 s (AcbC, 0 s), 169 ± 23 s (sham, 10 s), 179 ± 33 s (AcbC, 10 s), 231 ± 65 s (sham, 20 s), and 136 ± 37 s (AcbC, 20 s). Again, ANOVA revealed no effects of lesion or programmed delay and no interaction (maximum *F*_1,38 _= 1.61, NS). When the square-root-transformed number of responses on the inactive lever in session 10 was analysed with the experienced delays up to that point as a predictor, using the model lesion_2 _× experienced inactive-response-collection delay_cov _just as for the active lever analysis, there was no lesion × experienced delay interaction (*F *< 1, NS).

### Discrimination of relative reinforcer magnitude (Experiment 1B)

**Figure 8:**
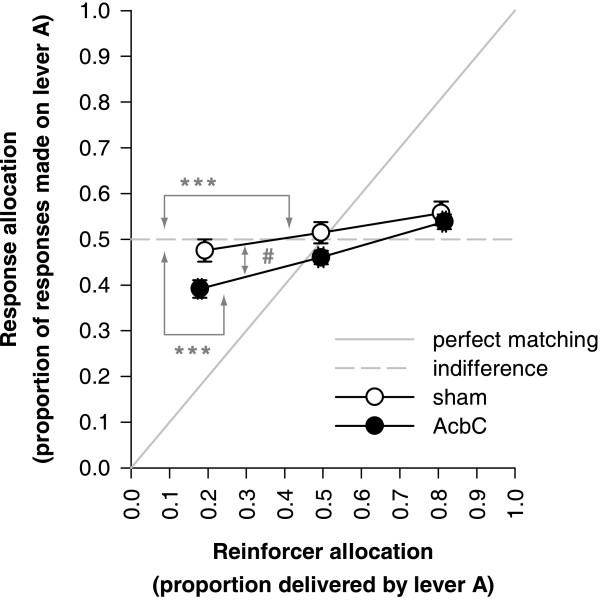
**Discrimination of reinforcer magnitude: matching of relative response rate to relative reinforcement rate **AcbC-lesioned rats exhibited better sensitivity to the difference between 1 and 4 food pellets than shams did. Subjects responded on two concurrent RI-60-s schedules, designated A and B, and the reinforcer magnitude for each schedule was varied. Data from the last session of each condition are plotted (sessions 11, 19, and 27; see Table 1); programmed reinforcement ratios were 0.2 (1 food pellet on schedule A and 4 pellets on schedule B), 0.5 (1:1 pellets), and 0.8 (4:1 pellets). The abscissa (horizontal axis) shows experienced reinforcement ratios (mean ± SEM); the ordinate (vertical axis) shows response allocation (mean ± SEM). Both groups exhibited substantial undermatching (deviation away from the predictions of the matching law and towards indifference). However, neither group was indifferent to the reinforcement ratio: the sham and AcbC groups both adjusted their response allocation towards the lever delivering the reinforcer with the greater magnitude (*** *p *< .001). Matching was better in AcbC-lesioned rats than in shams (lines of different gradient, # *p *= .021), suggesting that they were more sensitive to the difference between 1 and 4 food pellets.

Relative preference for two reinforcers may be inferred from the distribution of responses on concurrent variable interval schedules of reinforcement [31-33]. According to Herrnstein's matching law [31], if subjects respond on two concurrent schedules A and B delivering reinforcement at rates *r*_A _and *r*_B _respectively, they should allocate their response rates *R*_A _and *R*_B _such that *R*_A_/(*R*_A_+*R*_B_) = *r*_A_/(*r*_A_+*r*_B_). Overmatching is said to occur if subjects prefer the schedule with the higher reinforcement rate more than predicted by the matching law; undermatching is the opposite. Both sham-operated and AcbC-lesioned rats were sensitive to the distribution of reinforcement that they received on two concurrent random interval (RI) schedules, altering their response allocation accordingly. Subjects preferred the lever on which they received a greater proportion of reinforcement. In general, subjects did not conform to the matching law, but exhibited substantial undermatching; this is common [33]. AcbC-lesioned rats exhibited better matching (less undermatching) than shams (Figure 8), suggesting that their sensitivity to the relative magnitudes of the two reinforcers was as good as, or better than, shams'.

**Table 1:** Training and testing schedule for reinforcer magnitude matching task (Experiment 1B) Subjects were trained to respond on two levers (designated A and B) separately and then concurrently under interval schedules of reinforcement. In sessions 8–27, their preference for reinforcers of different magnitudes was assessed. The third column, labelled '*f*A', indicates the fraction of responses that would be allocated to lever A [i.e. A/(A+B)] were the subject to obey the matching law [31]. All concurrent (two-lever) schedules were subject to a 2 s changeover delay (COD), described in the Methods.

Day	Condition	*f*A	Lever A	Lever B
1	One-lever training	--	RI-2s, 1-pellet reinforcer	absent
2	One-lever training	--	absent	RI-2s, 1-pellet reinforcer
3	One-lever training	--	RI-15s, 1-pellet reinforcer	absent
4	One-lever training	--	absent	RI-15s, 1-pellet reinforcer
5	One-lever training	--	RI-30s, 1-pellet reinforcer	absent
6	One-lever training	--	absent	RI-30s, 1-pellet reinforcer
7	Two-lever training	0.5	RI-30s, 1-pellet reinforcer	RI-30s, 1-pellet reinforcer
8–11	1:1 magnitude	0.5	RI-60s, 1-pellet reinforcer	RI-60s, 1-pellet reinforcer
12–19	4:1 magnitude	0.8	RI-60s, 4-pellet reinforcer	RI-60s, 1-pellet reinforcer
20–27	1:4 magnitude	0.2	RI-60s, 1-pellet reinforcer	RI-60s, 4-pellet reinforcer

To analyse these data, the proportion of pellets delivered by lever A (see Methods), and the proportion of responses allocated to lever A, were calculated for each subject for the last session in each of the three programmed reinforcement distribution contingencies (session 11, programmed reinforcement proportion 0.5; session 19, programmed proportion 0.8; session 27, programmed proportion 0.2; see Table 1). The analysis used a model of the form response proportion = lesion_2 _× (experienced reinforcer distribution_cov _× S); the factor × covariate term was included in the model. Analysis of sham and AcbC groups separately demonstrated that both groups altered their response allocation according to the distribution of reinforcement, i.e. that both groups discriminated the two reinforcers on the basis of their magnitude (effects of reinforcer distribution; sham: *F*_1,47 _= 16.6, *p *< .001; AcbC: *F*_1,39 _= 97.2, *p *< .001). There was also a significant lesion × reinforcer distribution interaction (*F*_1,86 _= 5.5, *p *= .021), indicating that the two groups' matching behaviour differed, with the AcbC-lesioned rats showing better sensitivity to the relative reinforcer magnitude than the shams (Figure 8). These statistical conclusions were not altered by including counterbalancing terms accounting for whether lever A was the left or right lever (the left having been the active lever previously in Experiment 1A), or whether a given rat had been trained with 0, 10, or 20 s delays in Experiment 1A.

### Switching behaviour during concurrent schedule performance (Experiment 1B)

Because switching behaviour has the potential to influence behaviour on concurrent schedules e.g. [34], we also analysed switching probabilities. AcbC-lesioned rats were less likely than shams to switch between levers when responding on two identical concurrent RI schedules with a changeover delay (COD) of 2 s. Responses on the left and right levers were sequenced for sessions 8–11 (concurrent RI-60s schedules, each delivering a one-pellet reinforcer; see Methods and Table 1), and the probabilities of switching from one type of response to another, or repeating the same type of response, were calculated. The switch probabilities were analysed by one-way ANOVA; this revealed an effect of lesion (*F*_1,42 _= 8.88, *p *= .005). Mean switch probabilities (± SEMs) were 0.41 ± 0.02 (AcbC) and 0.49 ± 0.01 (sham).

### Effects of AcbC lesions on performance of a previously-learned instrumental response for delayed reinforcement (Experiment 2)

Due to mechanical faults, data from four subjects in session 10 (preoperative) and data from one subject in session 22 (postoperative) were not collected. Both sessions were removed from analysis completely, and data points for those sessions are plotted using the mean and SEM of the remaining unaffected subjects (but not analysed).

Preoperatively, the groups remained matched following later histological selection. Analysis of the last 3 preoperative sessions, using the model lesion intent_2 _× delay_3 _× (session_3 _× lever_2 _× S), indicated that responding was affected by the delays to reinforcement (delay: *F*_2,31 _= 5.46, *p *= .009; delay × lever: *F*_2,31 _= 19.5, *p *< .001), but there were no differences between the groups due to receive AcbC and sham lesions (terms involving lesion intent: maximum *F *was for session × lever × lesion intent, *F*_2,62 _= 1.844, NS). As expected, delays reduced the rate of responding on the active lever (*F*_2,31 _= 15.6, *p *< .001) and increased responding on the inactive lever (*F*_2,31 _= 8.12, *p *= .001) preoperatively.

**Figure 9:**
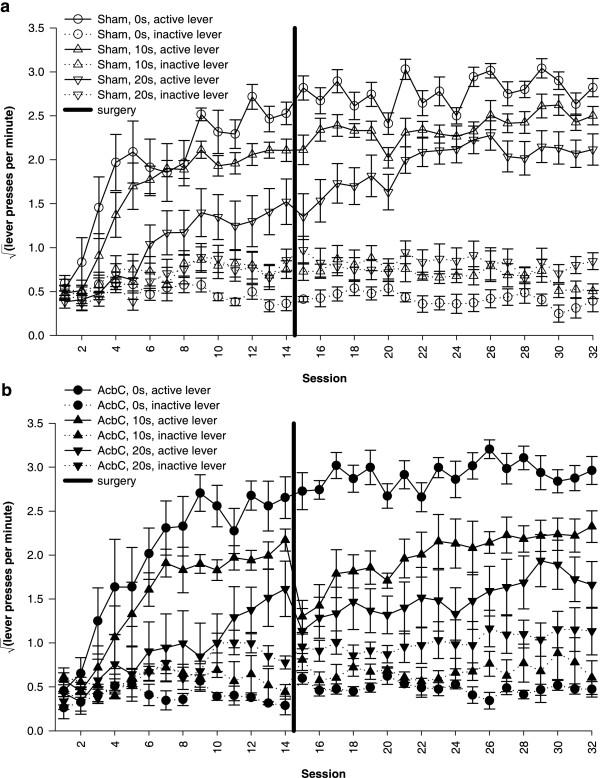
**Postoperative performance under an FR-1 schedule for delayed reinforcement **Data plotted to show the effects of delays. All groups discriminated between the active and the inactive lever, and delays retarded acquisition of the active lever response in both groups. Postoperatively, shams' performance was unaltered, as was that of AcbC-lesioned rats in the 0 s delay condition. However, active lever responding was impaired postoperatively in AcbC-lesioned rats in the 10 s and 20 s conditions. **(a) **Responding of sham-operated control rats, under all three response-reinforcer delay conditions. The vertical black line indicates the time of surgery, between testing sessions 14 and 15. **(b) **Responding of AcbC-lesioned rats under all delay conditions. The next figure replots these data to show the effect of the lesion more clearly.

**Figure 10:**
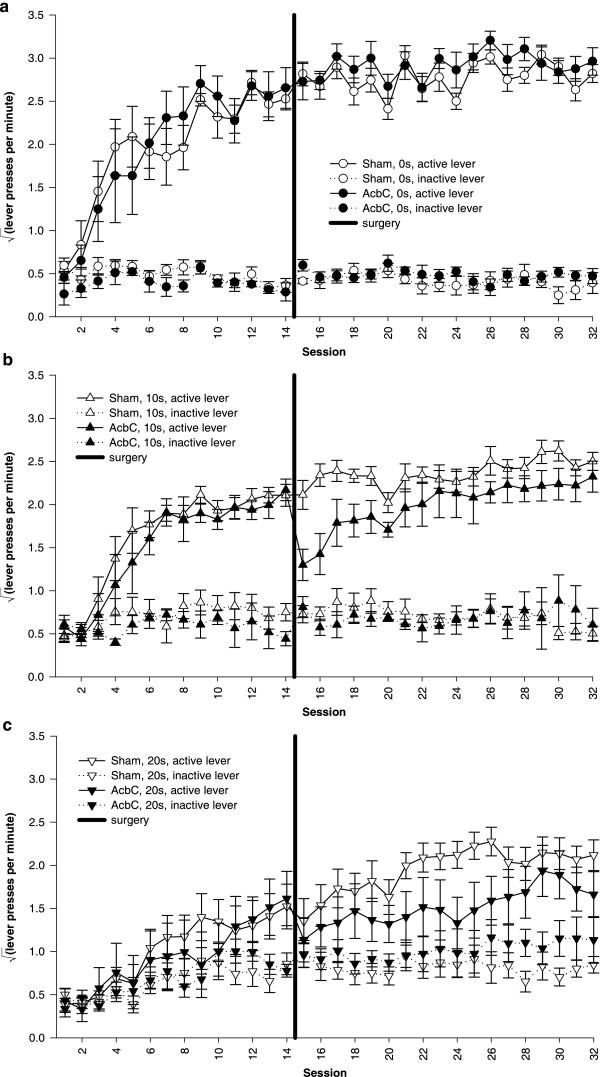
**Effect of AcbC lesions on performance of free-operant responding for delayed reinforcement **Data plotted to show the effects of AcbC lesions (same data as in the previous figure). There was a delay-dependent impairment in AcbC-lesioned rats, who were impaired by the lesion only when reinforcement was delayed. **(a) **With a delay of 0 s, AcbC-lesioned rats performed just as well as shams postoperatively. The vertical black line indicates the time of surgery, between testing sessions 14 and 15. **(b) **With a 10 s delay, AcbC-lesioned rats were impaired postoperatively compared to shams. **(c) **With a 20 s delay, the postoperative impairment in AcbC-lesioned rats was larger still, to the extent that their discrimination between active and inactive levers was no longer significant.

AcbC lesions selectively impaired performance of instrumental responding only when there was a response-reinforcer delay. There was no effect of the lesion on responding under the 0 s delay condition, but in the presence of delays, AcbC lesions impaired performance on the active lever (Figure 9; Figure 10). These conclusions were reached statistically as follows.

Subjects' responding on the relevant lever in the last preoperative session (session 14) was used as a covariate to increase the power of the analysis [35]. As expected, there were no significant differences in the covariates themselves between groups due to receive AcbC or sham surgery (terms involving lesion intent for the active lever: *F*s < 1, NS; for the inactive lever, lesion intent: *F*_1,31 _= 2.99, *p *= .094; lesion intent × delay: *F *< 1, NS). Analysis of the postoperative sessions, using the model lesion_2 _× delay_3 _× (session_17 _× lever_2 _× session-14-active-lever-responses_cov _× S), revealed a near-significant lesion × delay × session × lever interaction (*F*_22.4,335.5 _= 1.555,  = .699, *p *= .054). Furthermore, analysis of postoperative responding on the active lever, using the model lesion_2 _× delay_3 _× (session_17 _× session-14-active-lever-responses_cov _× S), revealed a session × delay × lesion interaction (*F*_17.3,259.5 _= 1.98,  = .541, *p *= .013) and a delay × lesion interaction (*F*_2,30 _= 3.739, *p *= .036), indicating that the lesion affected responding on the active lever in a delay-dependent manner. In an identical analysis of responding on the inactive lever (using inactive lever responding on session 14 as the covariate), no terms involving lesion were significant (maximum *F*: lesion, *F*_1,30 _= 1.96, *p *= .172), indicating that the lesion did not affect responding on the inactive lever.

Postoperatively, response-reinforcer delays continued systematically to decrease responding on the active lever, both in shams (Figure 9a; delay: *F*_2,20 _= 11.78, *p *< .001; session × delay: *F*_12.4,124.1 _= 2.36,  = .388, *p *= .008) and in AcbC-lesioned rats (Figure 9b; delay: *F*_2,11 _= 13.9, *p *= .001). Shams continued to discriminate between the active and inactive lever at all delays (lever: all groups *p *≤ .002; lever × session: all groups *p *≤ .003). AcbC-lesioned rats continued to discriminate at 0 s and 10 s (lever: *p *≤ .011; lever × session: *p *≤ .036), but AcbC-lesioned subjects in the 20 s condition failed to discriminate between the active and inactive levers postoperatively (lever: *F*_1,4 _= 1.866, *p *= .244; lever × session: *F *< 1, NS).

Lesioned subjects responded as much as shams at 0 s delay, but substantially less than shams at 10 s and 20 s delay (Figure 10). Again, analysis was conducted using responding on the relevant lever in session 14 (the last preoperative session) as a covariate. At 0 s, the lesion did not affect responding on the active lever (lesion: *F *< 1, NS; lesion × session: *F*_16,144 _= 1.34, NS). However, at 10 s, AcbC-lesioned rats responded significantly less than shams on the active lever (lesion: *F*_1,9 _= 7.08, *p *= .026; lesion × session: *F*_15.0,135.3 _= 3.04,  = .94, *p *< .001). Similarly, at 20 s, AcbC-lesioned rats responded less than shams on the active lever (lesion: *F*_1,10 _= 6.282, *p *= .031). There were no differences on responding on the inactive lever at any delay (*F*s ≤ 1.31, NS).

### Experienced response-delivery and response-collection delays (Experiment 2)

**Figure 11:**
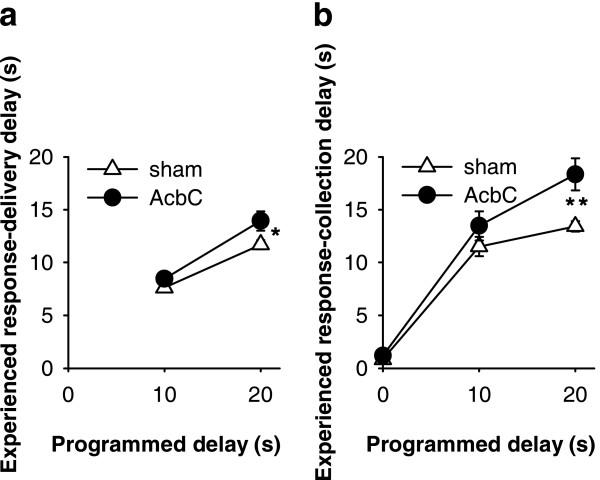
**Programmed and experienced delays to reinforcement following AcbC lesions made after initial training **AcbC-lesioned rats experienced slightly longer response-delivery and response-collection delays than shams in the 20 s condition. Lesions were made after initial training; postoperative experienced delays are plotted. (Compare Figure 6, in which rats had no preoperative experience of the task.) **(a) **Mean experienced response-delivery delays (one value calculated per subject). When the programmed delay was 0 s, reinforcers were delivered immediately so no data are shown. There were main effects of lesion (*F*_1,21 _= 9.14) and delay (*F*_1,21 _= 87.5, *p *< .001) but no lesion × delay interaction (*F*_1,21 _= 1.91, NS). When the programmed delay was 10 s, the experienced delays did not quite differ significantly between groups (*F*_1,10 _= 4.61, *p *= .057), but when the programmed delay was 20 s, AcbC-lesioned rats experienced longer response-delivery delays (*F*_1,11 _= 6.29, * *p *= .029). **(b) **Mean experienced response-collection delays (one value calculated per subject). There was a lesion × delay interaction (*F*_2,31 _= 3.85, *p *= .032), as well as main effects of lesion (*F*_1,31 _= 11.9, *p *= .002) and delay (*F*_2,31 _= 171, *p *< .001). AcbC-lesioned rats did not experience significantly different delays when the programmed delay was 0 s (*F*_1,10 _= 1.74, NS) or 10 s (*F*_1,10 _= 1.49, NS), but experienced significantly longer response-collection delays when the programmed delay was 20 s (*F*_1,11 _= 13.7, ** *p *= .003).

As in Experiment 1, AcbC-lesioned rats experienced the same response-delivery delays as shams when the programmed delay was 10 s, but experienced longer response-delivery delays when the programmed delay was 20 s (Figure 11a). Similarly, AcbC-lesioned rats experienced the same response-collection delays as shams when the programmed delay was 0 s, slightly but not significantly longer response-collection delays when the programmed delay was 10 s, and significantly longer response-collection delays when the programmed delay was 20 s (Figure 11b).

### Relationship between experienced delays and performance (Experiment 2)

**Figure 12:**
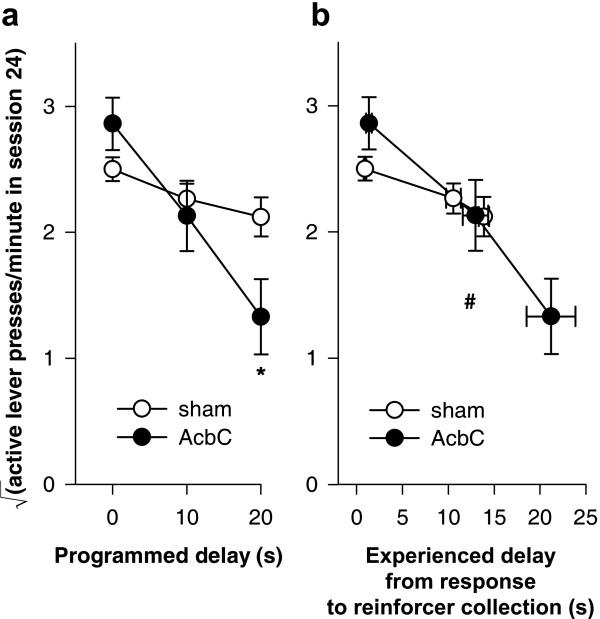
**Performance as a function of delays to reinforcement in animals trained preoperatively **Response-reinforcer delays systematically lowered the rate of free-operant instrumental responding, and this relationship was altered in AcbC-lesioned rats, even allowing for differences in response-collection delays experienced postoperatively. Lesions were made after initial training; postoperative experienced delays and response rates are plotted. (Compare Figure 7, in which rats had no preoperative experience of the task.) **(a) **The rate of responding on the active lever in session 24 (the 10^th ^postoperative session; compare Figure 7) is plotted against the programmed response-reinforcer delay. AcbC-lesioned rats responded significantly less than shams in the 20 s delay condition (* *p *= .025). **(b) **Responding on the active lever in session 24 (the 10^th ^postoperative session) plotted against the experienced response-to-reinforcer-collection delays for postoperative sessions up to and including session 24 (vertical error bars: SEM of the square-root-transformed number of responses in session 24; horizontal error bars: SEM of the experienced response-collection delay). The gradients of the two lines differed significantly (# *p *= .015; see text), indicating that the relationship between experienced delays and responding was altered in AcbC-lesioned rats, compared to sham-operated controls.

There was a systematic relationship between the postoperative response rate and the programmed delay of reinforcement, and this was altered in AcbC-lesioned rats. Figure 12a replots the rates of lever-pressing on session 24, the 10^th ^postoperative session (compare Figure 7). An analysis using the model lesion_2 _× programmed delay_3 _revealed a significant lesion × delay interaction (*F*_2,31 _= 5.09, *p *= .012). In this session, there was no significant effect of delays on shams' performance (*F*_2,20 _= 2.15, *p *= .143), though there was for AcbC-lesioned rats (*F*_2,11 _= 9.01, *p *= .005). There were no significant differences in responding on this session between shams and AcbC-lesioned rats in the 0 s condition (*F*_1,10 _= 3.10, *p *= .109) or the 10 s condition (*F *< 1, NS), but AcbC-lesioned rats responded less at 20 s delay (*F*_1,11 _= 6.74, *p *= .025).

Since the AcbC group experienced slightly longer response-delivery and response-collection delays than shams when the programmed delay was non-zero (Figure 11), as before, the rate of responding in session 24 was analysed as a function of the delays experienced postoperatively. The mean experienced response-collection delay was calculated for postoperative sessions up to and including session 24; the square-root-transformed number of lever presses in session 24 was then analysed using a general linear model of the form lesion_2 _× experienced delay_cov_, with the factor × covariate interaction term included in the model. This confirmed that the lesion affected responding in AcbC-lesioned rats, compared to controls, in a delay-dependent manner, over and above the postoperative differences in experienced delay (Figure 12b; lesion × experienced delay: *F*_1,33 _= 6.53, *p *= .015).

### Locomotor activity and body mass

**Figure 13:**
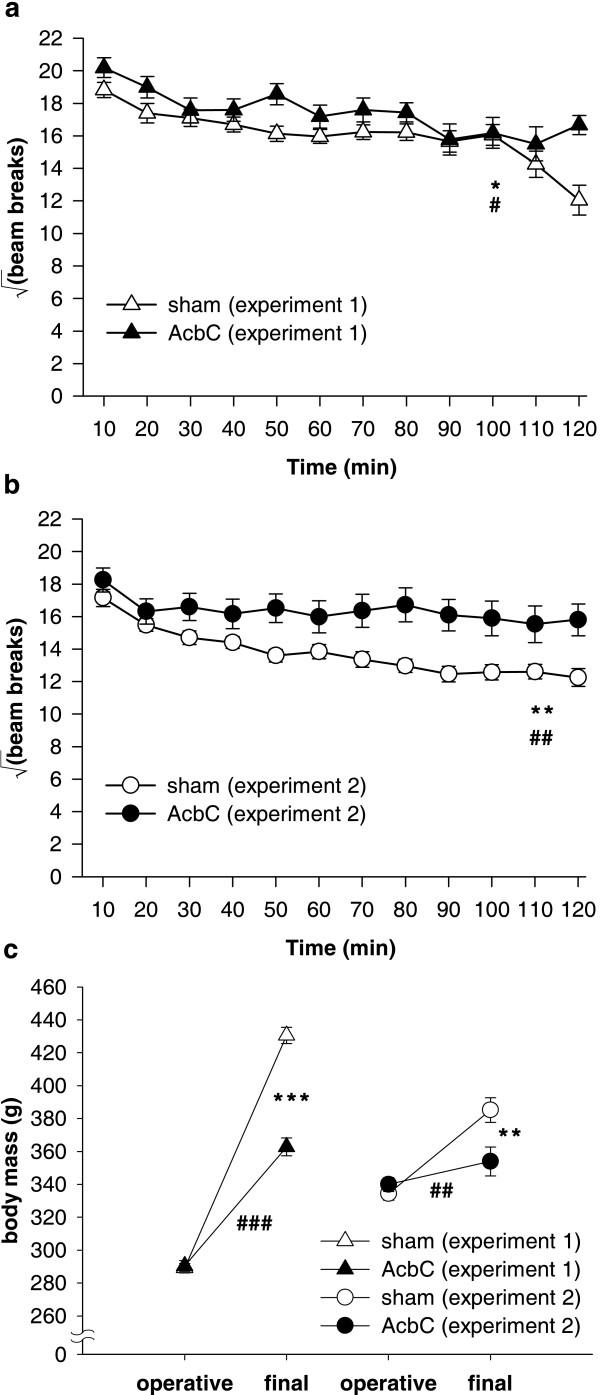
**Locomotor activity in a novel environment and body mass **AcbC-lesioned rats were significantly hyperactive compared to sham-operated controls, and gained less weight, in both Experiments 1 & 2. **(a) **Locomotor activity in Experiment 1. Analysis using the model lesion_2 _× (bin_12 _× S) revealed effects of lesion (*F*_1,42 _= 5.12, * *p *= .029), reflecting hyperactivity in the AcbC group, with additional effects of bin (*F*_5.7,237.9 _= 13.3,  = .515, *p *< .001), reflecting habituation, and a lesion × bin interaction (*F*_5.7,237.9 _= 2.52,  = .515, # *p *= .024). **(b) **Locomotor activity in Experiment 2. The same patterns were observed (data from five subjects were not recorded due to a mechanical error; lesion: *F*_1,37 _= 9.155, ** *p *= .004; bin: *F*_9.3,345.2 _= 13.5,  = .848, *p *< .001; lesion × bin: *F*_9.3,345.2 _= 3.18,  = .848, ## *p *= .001). **(c) **Preoperative and final body mass in both experiments. Preoperatively, masses did not differ between groups (Experiment 1: *F *< 1, NS; Experiment 2: *F*_1,42 _= 1.008, NS), but in both cases, AcbC-lesioned subjects gained less mass than controls (Experiment 1: lesion × time: *F*_1,41 _= 95.9, ### *p *< .001; group difference at second time point: *F*_1,42 _= 88.4, *** *p *< .001; Experiment 2: lesion × time: *F*_1,42 _= 13.53, ## *p *= .001; group difference at second time point: *F*_1,42 _= 7.37, ** *p *= .01).

AcbC-lesioned animals were hyperactive compared to sham-operated controls, and gained less mass then shams across the experiments (Figure 13), consistent with previous results [22,29,36].

## Discussion

These results establish that the AcbC contributes to learning of actions when the outcome is delayed. Lesions of the AcbC did not impair instrumental learning when the reinforcer was delivered immediately, but substantially impaired learning with delayed reinforcement, indicating that the AcbC 'bridges' action-outcome delays during learning. Lesions made after learning also impaired performance of the instrumental response in a delay-dependent fashion, indicating that the AcbC also contributes to the performance of actions for delayed reinforcement. Finally, the lesions did not impair the perception of relative reward magnitude as assessed by responding on identical concurrent interval schedules for reinforcers of different magnitude, suggesting that the impulsive choice previously exhibited by AcbC-lesioned rats [22] is attributable to deficits in dealing with delays to reinforcement.

### Effect of delays on instrumental learning in normal animals

Delays have long been known to retard instrumental learning [1,37]. Despite this, normal rats have been shown to acquire free-operant responding with programmed response-reinforcer delays of up to 32 s, or even 64 s if the subjects are pre-exposed to the learning environment [1]. Delays do reduce the asymptotic level of responding [1], though the reason for this phenomenon is not clear. It may be that when subjects learn a response with a substantial response-reinforcer delay, they never succeed in representing the instrumental action-outcome contingency fully. Alternatively, they may value the delayed reinforcer slightly less; finally, the delay may also retard the acquisition of a procedural stimulus-response habit and this might account for the decrease in asymptotic responding. It is not presently known to what degree responses acquired with a response-reinforcer delay are governed by declarative processes (the action-outcome contingency plus a representation of the instrumental incentive value of the outcome) or procedural mechanisms (stimulus-response habits), both of which are known to influence instrumental responding [38,39]; it is similarly not known whether the balance of these two controlling mechanisms differs from that governing responses learned without such a delay.

### Effect of AcbC lesions on instrumental learning and performance with or without delays

In the absence of response-reinforcer delays, AcbC-lesioned rats acquired an instrumental response normally, responding even more than sham-operated controls. In contrast, blockade of *N*-methyl-D-aspartate (NMDA) glutamate receptors in the AcbC has been shown to retard instrumental learning for food under a variable-ratio-2 (VR-2) schedule [in which *P*(reinforcer | response) ≅ 0.5] [40], as has inhibition or over-stimulation of cyclic-adenosine-monophosphate-dependent protein kinase (protein kinase A; PKA) within the Acb [41]. Concurrent blockade of NMDA and DA D1 receptors in the AcbC synergistically prevents learning of a VR-2 schedule [42]. Once the response has been learned, subsequent performance on this schedule is not impaired by NMDA receptor blockade within the AcbC [40]. Furthermore, infusion of a PKA inhibitor [41] or a protein synthesis inhibitor [43] into the AcbC *after *instrumental training sessions impairs subsequent performance, implying that PKA activity and protein synthesis in the AcbC contribute to the consolidation of instrumental behaviour. Thus, manipulation of Acb neurotransmission can affect instrumental learning. However, it is also clear that excitotoxic destruction of the AcbC or even the entire Acb does not impair simple instrumental conditioning to any substantial degree. Rats with Acb or AcbC lesions acquire lever-press responses on sequences of random ratio schedules [in which *P*(reinforcer | response) typically declines from around 1 to 0.05 over training] at near-normal levels [44,45]. In such ratio schedules, where several responses are required to obtain reinforcement, there is no delay between the final response and reinforcement, but there are delays between earlier responses and eventual reinforcement. It is therefore of interest that when differences between AcbC-lesioned rats and shams have been observed, AcbC-lesioned animals have been found to respond somewhat less than shams on such schedules late in training, when the ratio requirement is high [44,45], consistent with our present results. However, lesioned rats are fully sensitive to changes in the instrumental contingency [27,44,45]. Our present results indicate that when AcbC-lesioned rats are exposed to a FR-1 schedule for food [*P*(reinforcer | response) = 1] in the absence of response-reinforcer delays, they acquire the response at normal rates.

In contrast, when a delay was imposed between responding and reinforcement, AcbC-lesioned rats were impaired relative to sham-operated controls, in a systematic and delay-dependent fashion. The observation that learning was not affected at zero delay rules out a number of explanations of this effect. For example, it cannot be that AcbC-lesioned rats are in some way less motivated for the food *per se*, since they responded normally (in fact, more than shams) when the food was not delayed. Thus although the Acb and its dopaminergic innervation are clearly very important in motivating behaviour e.g. [23,46-48], this is not on its own a sufficient explanation for the present results. An explanation in terms of a rate-dependent impairment is also not tenable, since the AcbC-lesioned rats were capable (in the zero-delay condition) of responding at a level greater than they exhibited in the non-zero-delay conditions. Depletion of Acb DA also impairs rats' ability to work on high-effort schedules, where many, or very forceful, responses are required to obtain a given amount of food [47,48]. However, in the present experiments the ratio requirement (one response per reinforcer) and the force required per press were both held constant across delays, so this effect cannot explain the present results. Similarly, although AcbC lesions are known to impair the control over behaviour by Pavlovian conditioned stimuli e.g. [23,29,49-52], there was no Pavlovian stimulus that was differentially associated with delayed as opposed to immediate reinforcement in this task, so this cannot explain the present results.

Our results also indicated that when there were programmed delays to reinforcement, AcbC-lesioned animals experienced longer response-reinforcer collection delays, partly due to their failure to collect the reinforcer as promptly as shams. These additional experienced delays probably retarded learning. However, in addition to this effect, there was a further deficit exhibited by AcbC-lesioned rats: even allowing for the longer response-collection delays that they experienced, their instrumental learning was impaired more by delays than that of sham-operated controls. Deficits in learning with delayed reinforcement may account for some of the variability in the effect of AcbC lesions or local pharmacological manipulations on instrumental learning across different schedules.

The fact that pre-exposure to the context improves instrumental learning in normal rats [1] suggests one possible mechanism by which AcbC lesions might retard learning when delays are present. When a reinforcer arrives, it may be associated either with a preceding response, or with the context. Therefore, in normal animals, pre-exposure to the context may retard the formation of context-reinforcer associations by latent inhibition, or it might serve to retard the formation of associations between irrelevant behaviours and reinforcement. Similarly, non-reinforced exposure to the context forces the subjects to experience a zero-response, zero-reinforcer situation, i.e. *P*(*outcome *| *no action*) = 0. When they are then exposed to the instrumental contingency, such that *P*(*outcome *| *action*) > 0, this prior experience may enhance their ability to detect the instrumental contingency Δ*P *= *P*(*outcome *| *action*) - *P*(*outcome *| *no action*). In one aversive Pavlovian conditioning procedure in which a conditioned stimulus (CS) was paired with electric shock, AcbC lesions have been shown to impair conditioning to discrete CSs, but simultaneously to enhance conditioning to contextual (background) CSs [53], though not all behavioural paradigms show this effect [54,55]. It is therefore possible that enhanced formation of context-reinforcer associations may explain the retardation of response-reinforcer learning in AcbC-lesioned rats in the presence of delays.

The instrumental task used requires animals either to associate their response with the delayed food outcome (an action-outcome association that can be used for goal-directed behaviour), or to strengthen a stimulus-response association (habit) when the reinforcer eventually arrives [38,39]. Both mechanisms require the animal to maintain a representation of their past action so it can be reinforced (as a habit) or associated with food when the food finally arrives. This mnemonic requirement is not obviated even if the animal learns to predict the arrival of food using discriminative stimuli, and uses these stimuli to reinforce its responding (conditioned reinforcement): in either case, since the action precedes reinforcement, some trace of past actions or stimuli must persist to be affected by the eventual delivery of food.

A delay-dependent impairment was also seen when AcbC lesions were made after training. This indicates that the AcbC does not only contribute to the learning of a response when there is an action-outcome delay: it also contributes to the performance of a previously-learned response. Again, AcbC-lesioned rats were only impaired when that previously-learned response was for delayed (and not immediate) reinforcement. Of course, learning of an instrumental response depends upon the animal being able to perform that response; preventing an animal from pressing a lever (a performance deficit) would clearly impair its ability to learn an instrumental response on that lever to obtain food. In the present set of experiments, it is clear that AcbC-lesioned rats were just as able to perform the response itself (to press the active lever and to discriminate it physically from the inactive lever) as controls, as shown by their normal performance in the zero-delay condition, so it is not clear whether the delay-dependent impairments in learning and performance can be attributed to the same process. Again, since responding was unaffected in the zero-delay condition, many alternative interpretations (such as a lack of motivation to work for the food) are ruled out. It may be that AcbC-lesioned rats are impaired at representing a declarative instrumental action-outcome contingency when the outcome is delayed, or in forming or executing a procedural stimulus-response habit when the reinforcing event does not follow the response immediately. It may also be that they represent the action-outcome contingency normally but value the food less because it is delayed, and that this affects responding in a free-operant situation even though there is no alternative reinforcer available.

### Discrimination of reinforcer magnitude in AcbC-lesioned rats

Excitotoxic lesions of the whole Acb do not prevent rats from detecting changes in reward value (induced either by altering the concentration of a sucrose reward or by changing the deprivational state of the subject) [27]. Such lesions also do not impair rats' ability to respond faster when environmental cues predict the availability of larger rewards [28], and nor does inactivation of the Acb with local anaesthetic or blockade of AMPA glutamate receptors in the Acb [56]; the effects of intra-Acb NMDA receptor antagonists have varied [57,58]. AcbC-lesioned rats can still discriminate large from small rewards [24,25]. Similarly, DA depletion of the Acb does not affect the ability to discriminate large from small reinforcers [59-61], and systemic DA antagonists do not affect the perceived quantity of food as assessed in a psychophysical procedure [62]. Our study extends these findings by demonstrating that excitotoxic AcbC lesions do not impair rats' ability to allocate their responses across two schedules in proportion to the experienced reinforcement rate, even when the two schedules are identical except in the magnitude of the reinforcements they provide, thus demonstrating their sensitivity to reinforcer magnitude is quantitatively no worse than shams'. In this experiment, there was substantial undermatching, but this is common [33,63] see also [64,65]; differential cues signalling the two rewards might have improved matching but were not used in the present experiments since it is known that AcbC lesions can themselves affect rats' sensitivity to cues signalling reinforcement [23,29,49-52]. Given that AcbC-lesioned subjects showed a reduced probability of switching between two identical RI schedules, it may be the case that an enhanced sensitivity to the COD accounts for the better matching exhibited by the AcbC-lesioned rats [34]. Alternatively, the lesion may have enhanced reinforcer magnitude discrimination or improved the process by which behaviour allocation is matched to environmental contingencies. In summary, the present results suggest that AcbC damage leads to pathological impulsive choice (preferring a small, immediate reinforcer to a large, delayed reinforcer) [22] not through any relative lack of value of large reinforcers, but through a specific deficit in responding for delayed reinforcement.

### Contribution of the AcbC to reinforcement learning

The term 'reinforcement learning' simply means learning to act on the basis of reinforcement received; it is a term used in artificial intelligence research [66] that does not specify the mechanism of such learning [67,68]. Our present results indicate that the AcbC is a reinforcement learning structure that is critical for instrumental conditioning when outcomes are delayed, consistent with electrophysiological and functional neuroimaging evidence indicating that the ventral striatum responds to recent past actions [10,15] and to predicted future rewards [8-15], and with computational models suggesting a role for the striatum in predicting future primary reinforcement [20,21]. However, when reward is certain and delivered immediately, the AcbC is not necessary for the acquisition of instrumental responding. The delay-dependent role of the AcbC indicates that it plays a role in allowing actions to be reinforced by bridging action-outcome delays through a representation of past acts or future rewards. Acb lesions have also produced delay-dependent impairments in a delayed-matching-to-position task [69,70]; their effects on the delayed-matching-to-sample paradigm have also been studied, but a more profound and delay-independent deficit was observed, likely due to differences in the specific task used [71]. Finally, the AcbC is not alone in containing neurons that respond to past actions and future rewards. The dorsal striatum is another such structure [10,15,72,73]; expression of stimulus-response habits requires the dorsal striatum [74,75], and the rate at which rats learn an arbitrary response that delivers electrical stimulation to the substantia nigra is correlated with the degree of potentiation of synapses made by cortical afferents onto striatal neurons, a potentiation that requires DA receptors [76,77]. The prelimbic area of rat prefrontal cortex is important for the detection of instrumental contingencies and contributes to goal-directed, rather than habitual, action [78,79]. Similarly, the orbitofrontal cortex and basolateral amygdala encode reinforcement information and project to the AcbC, and lesions of these structures can produce impulsive choice see [24,80-82]. It is not yet known whether lesions of these structures also impair learning with delayed reinforcement.

## Conclusions

We have demonstrated that excitotoxic lesions of the AcbC do not prevent rats from learning a simple instrumental response when the reinforcing outcome follows their action immediately. However, AcbC lesions impair rats' ability to learn the same instrumental response when the outcome is delayed. The lesions also impair performance of an instrumental response that was learned preoperatively, but again only when response-reinforcer delays were present. These results suggest that the AcbC makes a specific contribution to reinforcement learning and instrumental performance when reinforcing outcomes do not arrive immediately but are delayed. AcbC dysfunction, which is known to promote impulsive choice, appears to cause rats to be temporally short-sighted, learning preferentially about the proximal consequences of their actions and preferring immediate over delayed rewards.

## Methods

### Overview of experiments

#### Experiment 1A: Effects of AcbC lesions on acquisition of instrumental responding with delayed reinforcement

Fifty naïve rats received excitotoxic lesions of the AcbC (*n *= 26) or sham lesions (*n *= 24). Two died postoperatively. Subjects were next trained in a task in which they had continuous access to two identical levers; one lever delivered a single food pellet each time it was pressed, and the other lever had no effect. For some rats, the food pellet was delivered immediately after the lever press (0 s condition; *n *= 8 AcbC-lesioned rats and 8 shams). For others, each pellet was delayed by either 10 s (8 AcbC, 8 sham) or 20 s (8 AcbC, 8 sham). Subjects were trained for 14 sessions.

#### Experiment 1B: Effects of AcbC lesions on the ability to match response distribution to reinforcer magnitude distribution

After the same rats had their locomotor activity assessed, they moved on to a task testing their ability to judge differences in the magnitude of two reinforcers. They were again offered two levers, but this time both levers delivered reinforcement on a variable-interval schedule, which provides reinforcement in an intermittent and temporally unpredictable fashion. Reinforcers consisted of either 1 or 4 sucrose pellets. Over sessions, the levers' roles changed so that the ratio of the sizes of the reinforcers available on the two levers was 4:1, 1:1, or 1:4. Subjects' responding was measured to establish their ability to judge the relative differences in reinforcer magnitudes and to allocate their responses according to the matching law [31-33]. Finally, they were killed and perfused for histology.

#### Experiment 2: Effects of AcbC lesions on performance of a previously-learned instrumental response for delayed reinforcement

A further 48 naïve rats were trained to acquire an instrumental response as before, with delays to reinforcement of 0 s (*n *= 16), 10 s (*n *= 16), or 20 s (*n *= 16). One rat spontaneously fell ill with a colonic volvulus and was killed. Once the subjects had been trained for 14 sessions, they were allocated to receive either AcbC lesions or sham surgery (0 s: 8 AcbC, 7 sham; 10 s: 8 AcbC, 8 sham; 20 s: 8 AcbC, 8 sham). Sham and AcbC groups were matched for performance preoperatively: within each delay condition, rats were ranked by their rates of responding on the active lever at the end of training, and rats with equivalent levels of performance were randomized to receive sham or AcbC lesion surgery. They were then retested postoperatively on the same task for a further 18 sessions (giving 32 sessions in total), with each rat experiencing the same delay as it had preoperatively. These rats then had their locomotor activity assessed, and were killed and perfused for histology.

### Subjects and housing conditions

Subjects were male Lister hooded rats (Harlan-Olac UK Ltd) housed in a temperature-controlled room (minimum 22°C) under a 12:12 h reversed light-dark cycle (lights off 07:30 to 19:30). Subjects were approximately 15 weeks old on arrival at the laboratory and were given a minimum of a week to acclimatize, with free access to food, before experiments began. Experiments took place between 09:00 and 21:00, with individual subjects being tested at a consistent time of day. Subjects had free access to water. During behavioural testing, they were maintained at 85–90% of their free-feeding mass using a restricted feeding regimen. Feeding occurred in the home cages at the end of the experimental day. All procedures were subject to UK Home Office approval (Project Licences PPL 80/1324 and 80/1767) under the Animals (Scientific Procedures) Act 1986.

### Excitotoxic lesions of the nucleus accumbens core

Subjects were anaesthetized with Avertin (2% w/v 2,2,2-tribromoethanol, 1% w/v 2-methylbutan-2-ol, and 8% v/v ethanol in phosphate-buffered saline, sterilized by filtration, 10 ml/kg i.p.) and placed in a Kopf or Stoelting stereotaxic frame (David Kopf Instruments, Tujunga, California, USA; Stoelting Co., Wood Dale, Illinois, USA) fitted with atraumatic ear bars. The skull was exposed and a dental drill was used to remove the bone directly above the injection and cannulation sites. The dura mater was broken with the tip of a hypodermic needle, avoiding damage to underlying venous sinuses. Excitotoxic lesions of the AcbC were made by injecting 0.5 μl of 0.09 M quinolinic acid (Sigma, UK) through a glass micropipette at coordinates 1.2 mm anterior to bregma, ± 1.8 mm from the midline, and 7.1 mm below the skull surface at bregma; the incisor bar was 3.3 mm below the interaural line [83]. The toxin had been dissolved in 0.1 M phosphate buffer (composition 0.07 M Na_2_HPO_4_, 0.028 M NaH_2_PO_4 _in double-distilled water, sterilized by filtration) and adjusted with NaOH to a final pH of 7.2–7.4. Toxin was injected over 3 min and the micropipette was left in place for 2 min following injections. Sham lesions were made in the same manner except that vehicle was infused. At the end of the operation, animals were given 15 ml/kg of sterile 5% w/v glucose, 0.9% w/v sodium chloride intraperitoneally. They were given a week to recover, with free access to food, and were handled regularly. Any instances of postoperative constipation were treated with liquid paraffin orally and rectally. At the end of this period, food restriction commenced or was resumed.

### Behavioural apparatus

Behavioural testing was conducted in one of two types of operant chamber of identical configuration (from Med Associates Inc, Georgia, Vermont, USA, or Paul Fray Ltd, Cambridge, UK). Each chamber was fitted with a 2.8 W overhead house light and two retractable levers on either side of an alcove fitted with an infrared photodiode to detect head entry. Sucrose pellets (45 mg, Rodent Diet Formula P, Noyes, Lancaster, New Hampshire, USA) could be delivered into the alcove. The chambers were enclosed within sound-attenuating boxes fitted with fans to provide air circulation. The apparatus was controlled by software written by RNC in C++ [84] using the Whisker control system [85].

### Instrumental conditioning with delayed reinforcement

A variety of free-operant schedules may be used to assess instrumental acquisition with delayed reinforcement [1]. We used the simplest possible free-operant schedule: each response scheduled a reinforcer after the programmed delay (Figure 1). In such a schedule, if the subject responds during the delay, the experienced response-reinforcer delay will not match the programmed delay (as the second response is temporally close to the first reinforcer). However, this schedule has the advantage that the response-reinforcer contingency is constant (every response does in fact cause the delivery of reinforcement) and the reinforcement rate is not constrained [1]. So that responding could be attributed to the instrumental response-reinforcer contingency, rather than the effects of general activity or reinforcement itself, responding on the active lever was compared to responding on a control lever that had no programmed consequence. Different groups of lesioned and sham-operated subjects were trained using different delays; the delay was consistent for every subject. Delays of 0, 10, and 20 s were used.

Alternative free-operant schedules for this purpose exist, such as one in which the first response sets up reinforcement, and a subsequent response made before the reinforcer is delivered postpones reinforcement, in order to keep the delay between the last response and the reinforcer constant (known as a tandem fixed-ratio-1 differential-reinforcement-of-other-behaviour or FR-1-DRO schedule). However, the tandem FR-1-DRO schedule constrains the maximum rate of reinforcement, which also decreases as the delay being used increases. Furthermore, it does not hold constant the probability of reinforcement given a response, and it introduces two opposing contingencies: some responses make reinforcement more likely, while others (those during the delay) make it less likely [1]. Therefore, we did not use this schedule. Similarly, the acquisition of instrumental responding with delayed reinforcement may be assessed with discrete-trial tasks. For example, two levers could be presented in trials occurring at fixed intervals, the levers could be retracted when a response had been made, and responding on one lever could be reinforced after a delay, taking care to avoid a differential Pavlovian contingency between presentation or retraction of one lever and reinforcement, since responding might then be due to Pavlovian conditioning autoshaping; [86,87] rather than the instrumental contingency. However, this discrete-trial schedule would also divide up the session explicitly into response-food delays and food-response (intertrial) times, a process that might aid learning and/or be affected by the lesion. Furthermore, there is prior evidence that AcbC lesions impair rats' ability to choose a delayed reward over an immediate reward in the discrete-trial situation [22]. Therefore, to address the more general question of whether the AcbC is required to acquire instrumental responding with delayed reinforcement, we chose instead to use a free-operant schedule; this seemed to us to mimic best the real-life problem of relating actions to their outcomes with no explicit demarcation of when a response had been made or when a response was permissible.

Immediately after subjects were placed in the operant chamber, the sessions began. The houselight was illuminated, and remained on for each 30-min session. Two levers were extended into the chamber. All lever responses were first 'debounced' to 10 ms (i.e. if a response occurred within 10 ms of a previous valid response it was attributed to mechanical bounce and ignored). Other than this, all lever presses and nosepokes into the food alcove were recorded. Responding on the left (active) lever caused a single pellet to be delivered following a delay, under a fixed-ratio-1 (FR-1) schedule (Figure 1). To attribute acquisition of a lever-press response to the instrumental contingency, it is also necessary to control for the effects of reinforcer delivery itself [1]; therefore, responding on the active lever was compared to responding on the right (inactive) lever, which had no programmed consequence. To minimize any potential contribution of conditioned reinforcement to the task, no explicit signals were associated with pellet delivery other than the noise of the pellet dispenser apparatus.

### Locomotor activity in a novel environment

Since general activity levels might influence instrumental responding, locomotor activity was also measured, using wire mesh cages, 25 (W) × 40 (D) × 18 (H) cm, equipped with two horizontal photocell beams situated 1 cm from the floor that enabled movements along the long axis of the cage to be registered. Subjects were placed in these cages, which were initially unfamiliar to them, and their activity was recorded for 2 h. All animals were tested in the food-deprived state. Locomotor hyperactivity and reduced weight gain have previously been part of the phenotype of AcbC-lesioned rats, though without alterations in the consumption of the reinforcer used in the present experiments [22,29,36].

### Matching of response distribution to reinforcer magnitude distribution on a concurrent schedule

Subjects were trained in 30-min sessions to respond on both levers separately under interval schedules of reinforcement. The two levers were designated A and B; these were counterbalanced left/right (thus, for half the subjects in each group, lever A was the lever reinforced previously in the delay task; for the other half, it was the lever previously unreinforced). As before, responses were debounced to 10 ms. Training and testing proceeded according to Table 1. Random-interval-*x*-second (RI-*x*) schedules were implemented by having a clock tick once a second; each tick set up reinforcement with a probability *p *= 1/*x*. Once reinforcement had been set up for a schedule, the next response caused reinforcement to be delivered. Multiple pellets were delivered 0.5 s apart. For concurrent RI schedules, a 2 s changeover delay (COD) was imposed to discourage frequent switching between schedules [32-34,88]. The COD was implemented as follows: if a subject pressed lever B, it could only be reinforced if more than 2 s had elapsed since it last pressed lever A (and vice versa). The RI schedules could still set up reinforcement during the COD, but the subject could not earn that reinforcement until the COD had elapsed.

### Histology

Rats were deeply anaesthetized with pentobarbitone sodium (200 mg/ml, minimum of 1.5 ml i.p.) and perfused transcardially with 0.01 M phosphate-buffered saline (PBS) followed by 4% paraformaldehyde in PBS. Their brains were removed and postfixed in paraformaldehyde before being dehydrated in 20% sucrose for cryoprotection. The brains were sectioned coronally at 60 μm thickness on a freezing microtome and every third section mounted on chromium potassium sulphate/gelatin-coated glass microscope slides and allowed to dry. Sections were passed through a series of ethanol solutions of descending concentration (3 minutes in each of 100%, 95%, and 70% v/v ethanol in water) and stained for ~5 min with cresyl violet. The stain comprises 0.05% w/v aqueous cresyl violet (Raymond A. Lamb Ltd, Eastbourne, UK), 2 mM acetic acid, and 5 mM formic acid in water. Following staining, sections were rinsed in water and 70% ethanol before being differentiated in 95% ethanol. Finally, they were dehydrated and delipidated in 100% ethanol and Histoclear (National Diagnostics, UK) before being cover-slipped using DePeX mounting medium (BDH, UK) and allowed to dry. The sections were used to verify cannula and lesion placement and assess the extent of lesion-induced neuronal loss. Lesions were detectable as the absence of visible neurons (cell bodies of the order of 100 μm in diameter with a characteristic shape and appearance), often associated with a degree of tissue collapse (sometimes with consequent ventricular expansion when the lesion was adjacent to a ventricle) and gliosis (visible as the presence of smaller, densely-staining cells).

### Data analysis

Data collected by the chamber control programs were imported into a relational database (Microsoft Access 97) for case selection and analysed with SPSS 11. Figures were created with SigmaPlot 2001/v7 and Adobe Illustrator 8. All graphs show group means and error bars are ± 1 standard error of the mean (SEM) unless otherwise stated. Count data (lever presses and locomotor activity counts), for which variance increases with the mean, were subjected to a square-root transformation prior to any analysis [35]. Homogeneity of variance was verified using Levene's test [89]. General linear models are described as *dependent variable = A*_2 _× *B*_*cov *_× (*C*_5 _× *D*_*cov *_× *S*) where A is a between-subjects factor with two levels, B is a between-subjects covariate, C is a within-subjects factor with five levels, and D is a within-subjects covariate; S denotes subjects in designs involving within-subjects factors [90]. For repeated measures analyses, Mauchly's test of sphericity of the covariance matrix was applied [91] and the degrees of freedom corrected to more conservative values using the Huynh-Feldt epsilon  for any terms involving factors in which the sphericity assumption was violated [92].

## List of abbreviations used

, Huynh-Feldt epsilon

Acb, nucleus accumbens

AcbC, nucleus accumbens core

AcbSh, nucleus accumbens shell

AMPA, α-amino-3-hydroxy-5-methyl-4-isoxazolpropionate

ANCOVA, analysis of covariance

ANOVA, analysis of variance

COD, changeover delay

DA, dopamine

DRO, differential reinforcement of other behaviour

FR, fixed ratio

i.p., intraperitoneal

h, hour

min, minute

NMDA, *N*-methyl-D-aspartate

*P*(A), probability of event A occurring

*P*(A | B), probability of A occurring, given that B has occurred

PBS, phosphate-buffered saline

PKA, protein kinase A (cyclic-adenosine-monophosphate-dependent protein kinase)

RI, random interval

SEM, standard error of the mean

VR, variable ratio

v/v, volume per unit volume

w/v, weight per unit volume

## Authors' contributions

RNC conceived and designed the studies, supervised THCC, wrote the software, and drafted the manuscript. THCC participated in the design of the studies and tested the animals. The work contributed to THCC's MPhil thesis. Both authors performed surgery, processed histological material, analysed the results, and read and approved the final manuscript.
